# Dentistry in France

**Published:** 1854-04

**Authors:** 


### ARTICLE XVI.

### *Dentistry in France.*

rance, in sober minded England, as unhappily in America, the interests of our profession have been seriously compromised, from its sacred domains having been invaded by hordes of illiterate and unprincipled charlatans. Not a few people, imposed on by such persons, look upon our science as hardly deserving the name; and the proportion of quacks is so great, compared with the number of scientific, conscientious men, that the very name of dentist is with many a name, if not of dishonor, at least of discredit. By many, in fact, the science of dentistry would be described as "the knack of pulling out and putting in teeth," and a man who has acquired a little skill in these operations, and, by dint of lead and brass, can bungle through certain others, passes current as a master of the art. I am inclined to think this is peculiarly so in France. The number of self-celebrated dentists there, is enormous—composing a perfect legion of *Dis*Honor. You see their flaming advertisements, bristling with the teeth of their victims—like Indian wigwams hung with the scalps of the enemy—everywhere.

On the broad, beautiful Boulevards, in all the splendid arcades and spacious squares, are displayed showy frames, exhibiting full sets of artificial teeth, opening and shutting as teeth never opened and shut before, from morning to night; and grinning at the green and gullible public with a pertinacity and impudence only too characteristic of their inventors.

Dentists' saloons, showy as shaving saloons, (and in some respets not unlike them,) are rigged on wheels and dragged through the streets by gaily caparisoned horses, while the spirited proprietor sits in state by the side of the driver, and, as the vehicle stops from time to time, harangues the multitude very much after the fashion of our equally enterprising, but I trust more scrupulous, Connecticut pedlers. The harangue finished, the "wooden nutmegs"—I beg pardon—the patent teeth duly exhibited, and the public, without distinction, being invited to walk in, one of the hired *attaches* of the establishment, with his excruciating face half concealed by a dirty handkerchief, enters the saloon, and in a moment after returns, grinning like a clown, and informing the bystanders that whereas five minutes ago he had the horridest toothache in the world, he is now entirely cured and is the happiest man alive. Then one of his colleagues enters, and after a few moments of awful silence, makes his exit, swearing that meanwhile his impudent jaws have been circled with thirty-two as fine teeth as ever cracked a nut. These decoy ducks having "acted well their part," the success of the trick is seen by the fact that crowds of real sufferers now enter the gilded cage, (perhaps I should say the lion's mouth,) whence, it is needless to add, they are glad to emerge on any terms.

There are other tricks of the trade, too well known to every one who has visited France, and almost too ridiculous to mention. The latest appears in the form of a book, calling upon every man to pull and plug his own teeth, as if every man who attempted such a thing were not sure to have a fool for dentist.—***Dent. News Letter.***

